# The effectiveness of adjunctive measures in managing peri-implant mucositis: an umbrella review

**DOI:** 10.1186/s40729-022-00426-2

**Published:** 2022-06-08

**Authors:** Sompol Chuachamsai, Aneesha Acharya, Kai Fischer, Luigi Nibali, Dominic Ho, Georgios Pelekos

**Affiliations:** 1 Division of Periodontology and Implant Dentistry Faculty of Dentistry, The University of Hong Kong, Hong Kong, SAR China; 2 Dr D. Y. Patil Dental College and Hospital, Dr D. Y. Patil Vidyapeeth, Pune, India; 3 Clinic of Conservative and Preventive Dentistry, Division of Periodontology and Peri-Implant Diseases, University of Zurich, Zurich, Switzerland; 4 Periodontology Unit, Faculty of Dentistry, Oral and Craniofacial Sciences, Centre for Host-Microbiome Interactions, King’s College London, London, UK; 5 Division of Periodontology and Implant Dentistry, Faculty of Dentistry, The University of Hong Kong, Hong Kong, SAR China; 6 Department of Periodontology, Faculty of Dentistry, The University of Hong Kong, Prince Philip Dental Hospital, 34 Hospital Road, Sai Ying Pun, Hong Kong, 999077 SAR China

## Abstract

**Objectives:**

The purpose of this umbrella review was to gather and summarize the data from published systematic reviews (SRs) that compared non-surgical mechanical debridement (NSMD) with and without the use of adjunctive treatments on the management of peri-implant mucositis (PIM).

**Materials and methods:**

A protocol was developed and registered in PROSPERO (CRD42021254350) before the systematic search for the SRs. Seven electronic databases, including Cochrane Library, Embase (via Ovid), MEDLINE (via Pubmed), Proquest, Prospero, Scopus and Web of Science, were searched for published reviews. The search for unpublished and informally published reviews was further attempted in the last four databases. The methodological quality of the included reviews was assessed using AMSTAR 2.

**Results:**

Twelve included SRs assessed clinical studies published between 2014 and 2020, including a total of seventeen primary clinical trials. All SRs summarized data from individual studies and provided a narrative conclusion regarding the effectiveness of the adjunctive treatments. Only six SRs performed a meta-analysis (MA) of additional benefits of the adjunctive therapy for PIM, with results indicating no significant difference between the different treatment modalities. The overall confidence was adjudged ranging from critically low to low using AMSTAR 2 and significant additional benefits of any adjunctive treatments in comparison with NSMD were not apparent.

**Conclusion:**

Overall, the reviewed evidence did not support the use of adjunctive treatments for improvement of clinical outcomes in PM management as compared to NSMD alone.

**Supplementary Information:**

The online version contains supplementary material available at 10.1186/s40729-022-00426-2.

## Introduction

Peri-implant mucositis (PIM) is defined as inflammation of peri-implant mucosa without evidence of continuing marginal bone loss after initial bone remodeling [1], whereas peri-implantitis is defined as inflammation of peri-implant mucosa and additional marginal bone loss after initial bone healing [2]. Bacterial biofilm is the primary etiological agent in peri-implant mucositis and peri-implantitis [3, 4]. Considering that peri-implant mucositis precedes peri-implantitis, treatment of peri-implant mucositis is considered as the primary preventive modality for peri-implantitis [5]. The resolution of peri-implant mucositis may be achieved effectively by professional non-surgical mechanical debridement (NSMD) and enhanced oral hygiene practice (EOH) [6]. In addition, adjunctive treatments, including air-polishing, laser and photodynamics, antiseptics, and antibiotics, have been proposed to improve the outcomes of this treatment. Several systematic reviews (SRs) and meta-analyses (MA) have analyzed the effect of various adjunctive treatments compared to NSMD alone.

SRs provide a comprehensive synthesis of all available evidence (clinical studies) related to a specific intervention. This type of evidence synthesis focuses on a narrow review question, typically of direct comparison between two therapies and provides the highest level of evidence to designate health care decisions [7]. However, with the increasing number of systematic reviews and meta-analyses, treatment decisions can entail reading several systematic reviews. Therefore, it is appropriate to conduct an overview of reviews or "umbrella review" to compile data from multiple systematic reviews to support health care decision-making [8].

Umbrella reviews use a systematic method similar to systematic reviews, to compile information from systematic reviews instead of individual studies. In addition, umbrella reviews may examine different interventions for a particular disease, while systematic reviews usually focus on a single intervention. The comparison of similar systematic reviews can indicate the consistent or conflicting nature of evidence [9] and addresses the knowledge gap in available evidence for future research [10]. The present umbrella review aimed to gather and summarize the data from published systematic reviews that compared NSMD with adjunctive treatments and NSMD alone for managing PIM. The focus questions of the present umbrella review were:

1. What is the effectiveness of the NSMD with adjunctive measures compared with NSMD alone in managing PIM?
2. What is the quality of the systematic reviews concerning the effectiveness of adjunctive treatment in managing PIM?

## Material and methods

The protocol was developed and registered on PROSPERO (CRD42021254350) before the systematic search for the systematic reviews. The SRs included in this umbrella review reported a comparison of the effectiveness of NSMD combined with adjunctive interventions versus NSMD alone. The eligibility criteria and search strategy were constructed with the aid of the following PICOS elements:

- Population—adult patients with the diagnosis of peri-implant mucositis
- Intervention—non-surgical mechanical debridement with adjunctive interventions
- Comparison—non-surgical mechanical debridement alone
- Outcomes—clinical, microbiological and immunological parameters
- Study design—systematic reviews with or without meta-analysis.

### Search strategy

Seven electronic databases, including Cochrane Library, Embase (via Ovid), MEDLINE (via Pubmed), Proquest, Prospero, Scopus and Web of Science, were identified for published reviews. The search for unpublished and informally published reviews was further attempted in the last four databases. The search term "(peri-implant OR periimplant) AND (mucositis OR disease* OR infect* OR inflammation) AND (treatment OR therapy* OR intervention OR management OR managing OR instrumentation OR "plaque removal" OR intervention)" was used to search for title, abstract and keywords when applicable. Two search strategies were applied for each database. The first strategy was the keyword search with a document-type filter for reviews or systematic reviews. The second strategy was the keyword search without a document-type filter, but the additional "systematic review" term was incorporated into the original search term. Combining two search strategies ensures comprehensiveness of the results since the search with a document-type filter might be of limited sensitivity, and some electronic databases such as Embase could not provide a search filter to identify systematic reviews successfully [11]. In addition, the search was restricted to the English language. All the seven electronic databases were searched for relevant reviews with a publication date until September 15th, 2021. The references, journal title, study title, authors, years of publication and abstract of searched results were exported to an EndNote library (using the management software EndNote X9.3.3, macOS Big Sur). Any duplication was removed before constructing the final list for review selection.

### Review selection and additional searches

The review selection included two steps. The first step was screening the reviews by assessing the title and abstract. The second step involved screening by appraising the full text using the table of eligibility criteria (Additional file 1: Table S1). All steps were performed independently, by two reviewers (SC and AA). Any disparity was settled down by consensus and consultation with the third independent person in the team (GP).

The eligible systematic reviews were required to include the primary studies of adult patients diagnosed with peri-implant mucositis. The SRs that exclusively appraised treatment for peri-implantitis were excluded. The included systematic reviews summarized the outcome of primary studies and may also synthesize the data using descriptive analysis or meta-analysis. The primary studies included in the potential SRs were assessed against PICO elements using the same table of eligibility criteria (Additional file 1: Table S1). The irrelevant studies were identified and excluded. The SRs included in this umbrella review contain a minimum of one eligible primary study.

Cohen's k statistic was used to calculate an agreement between two reviewers. The inter-rater agreement for the title and abstract screening was 99.84%, and the Cohen kappa value was 0.97. The inter-rater agreement for full-text selection was 95%, and the Cohen kappa value was 0.89. In addition, hand-searching of the reference lists of the included systematic reviews was carried out to identify additional systematic reviews relevant to the PICO framework of this umbrella review.

### Data collection

One of the reviewers (SC) performed the data collection systematically. The data were entered directly into the spreadsheet and checked by the other reviewer (AA). Any disparity in data extraction was resolved by consensus. All included SRs were extracted for the data on the general characteristics of the reviews, clinical and methodological characteristics, synthesized results and conclusion. In addition, the data of the primary studies reported in the selected systematic reviews were also extracted for bibliographic details, clinical and methodology characteristics, result and conclusion, and quality assessment (risk of bias). The data items of the systematic reviews and primary studies are listed in Additional file 1: document 1.

The data were cross-checked with the original articles or the other systematic reviews to correct any reporting errors or completing the required information when the SR report was unclear. In cases where the original reviewers did not provide the overall risk-of-bias of each primary study, the suggested algorithm in the Cochrane handbook for systematic reviews of interventions was applied [12]. The risks were summarized as low, unclear or high based on the presence of the greatest risk in the key domains within the individual studies.

### Assessment of methodological quality of systematic reviews

The SRs included in the present umbrella review were assessed for the quality of methodology using AMSTAR 213. AMSTAR 2 is widely used to identify the quality of systematic reviews that include randomized or non-randomized trials of healthcare interventions. The overall confidence of each SR was determined based on the flaws or weaknesses in seven critical and nine non-critical domains [13]. The overall confidence of a systematic review was high when there was none of the critical flaw or only one non-critical flaw. The overall confidence was moderate when there was no critical flow or more than one non-critical flaw. The overall confidence was low when there was one critical flaw, and the overall confidence was critically low when there was more than one critical flaw. The assessment was performed independently by two reviewers (SC and AA). Any disparity in the assessment was settled by consensus.

### Data synthesis

Most of the SRs did not provide a definitive conclusion concerning the effect on the outcome measurement (e.g., bleeding on probing or probing depth of peri-implant sulcus) but tabulated the data from the included primary studies, a decision was made to apply the vote-counting method and present the ratio of the primary studies of each outcome parameter to illustrate the outcome of the available evidence in each systematic review.

An additional conclusion was further made based on the ratio of the primary studies [14]. A minimum of three primary studies in each SR was required to conclude the effect of the adjunctive interventions. The adjunctive treatment was considered an additional benefit if more than two-thirds of the primary studies presented significant positive results.

## Results

### Description of included systematic reviews

**Fig. 1:**
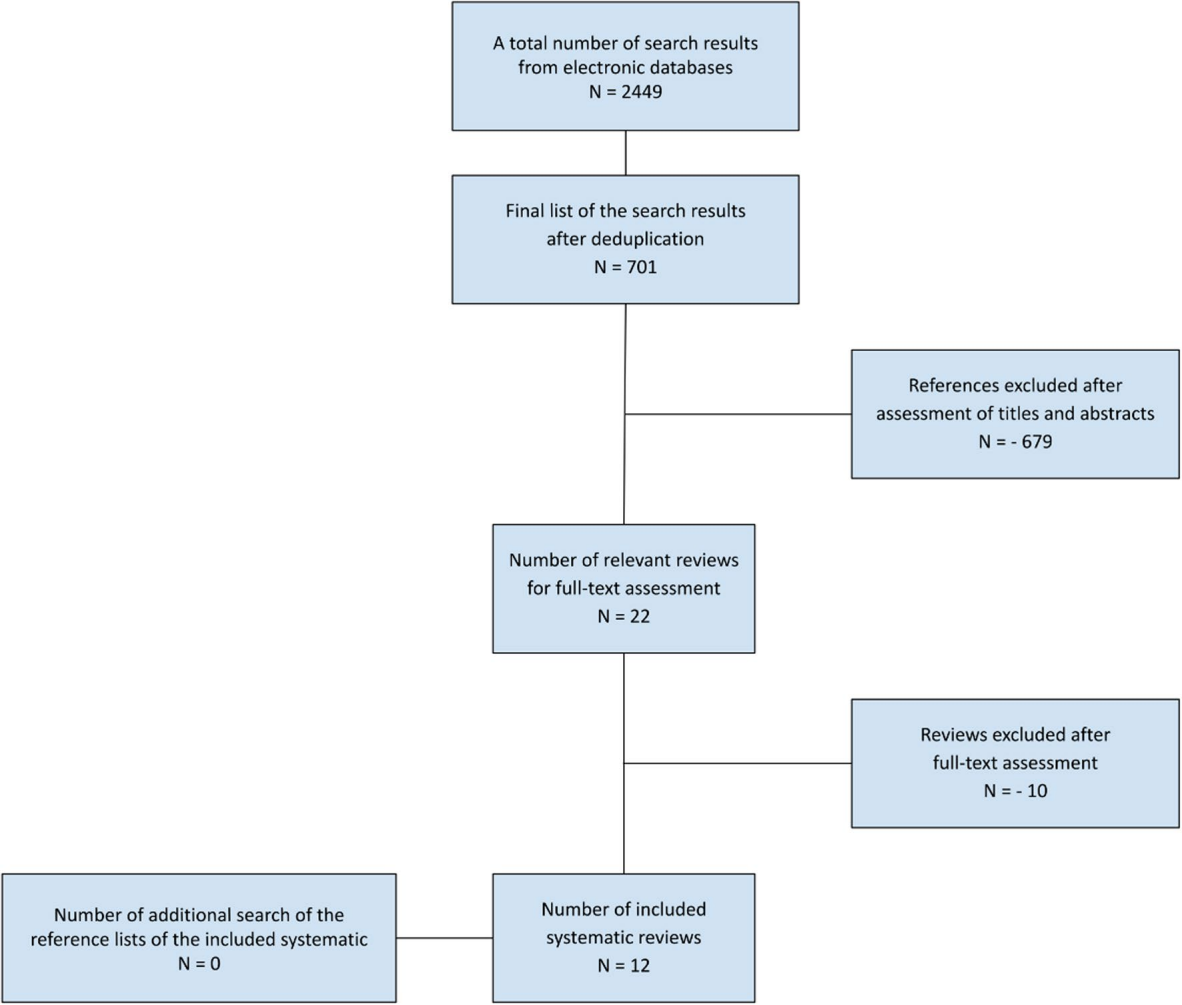
Flow diagram of the review selection process

The final list of 701 search results was constructed after de-duplication. The title and abstract screening resulted in the exclusion of 679 references. Out of the potential 22 references for full-text screening, ten references were excluded after assessing the full text. The reasons for the exclusion of each study are presented in Additional file 1: Table S2. Twelve SRs were included in the present umbrella review. All were published outside the Cochrane Database of Systematic Reviews (CDSR) from 2015 to 2020. The flow diagram of the review selection process is illustrated in Fig. 1.

The PICO frameworks in the included SRs differed. Some included populations with PIM and peri-implantitis or compared different adjunctive treatments. Among the included SRs, only one had a specific focus population and adjunctive treatment [15], analyzing adjunctive laser and photodynamic therapy for PIM. Eight SRs reviewed a specific adjunctive intervention for both peri-implant mucositis and peri-implantitis, which encompassed antiseptics [16, 17] probiotics [18–20] air-polishing [21], and laser and photodynamic treatment [22, 23]. Two SRs [24, 25] reviewed all adjunctive treatments for PIM, and one [26] reviewed all adjunctive intervention for both PIM and peri-implantitis. The summary of bibliographic information and the PICOS frameworks of included SRs are presented in Table 1.

**Table 1:**
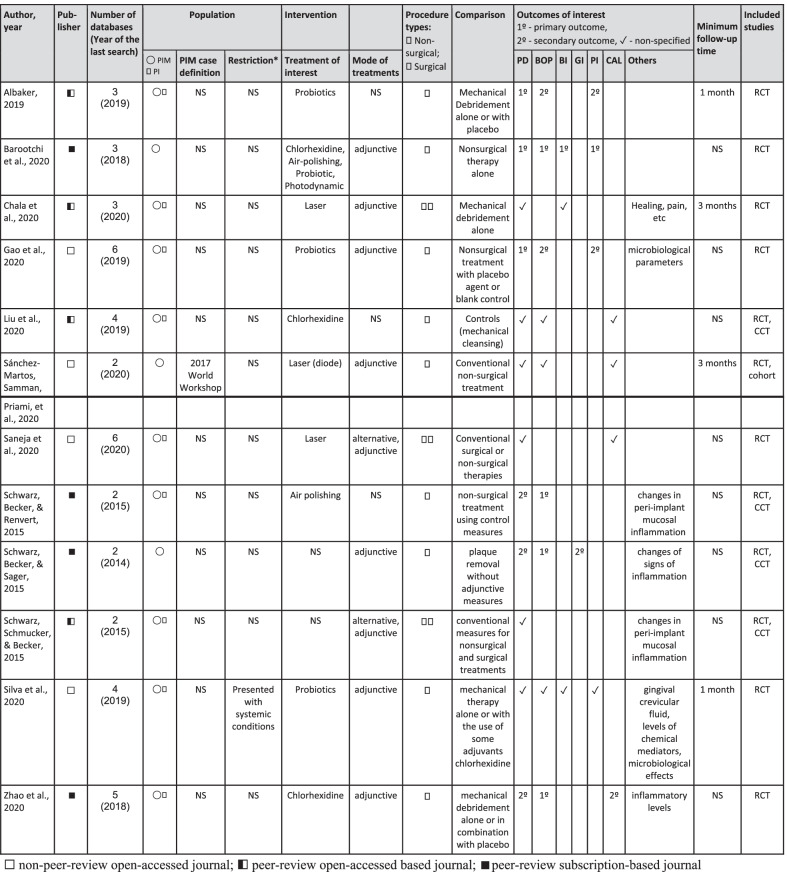
Characteristic of included systematic reviews

*NS* not specified in text, *PIM*  peri-implant mucositis, *PI* peri-implantitis, *RCT*  randomized controlled clinical trials, *CCT*  controlled clinical trials

^*^Restriction of systematic factors (i.e., diabetes, radiotherapy, smoking), history of periodontitis, or other patient factors

The assessment of each AMSTAR 2 criteria and the overall confidence of the systematic reviews are presented in Table 2. Affected by critical flaws, seven systematic reviews were scored as "low" in overall confidence, while the other five were scored as "critically low". The most common critical flaw was the lack of interpretation of the risk of bias when discussing the result, in nine systematic reviews [15, 18, 21–26]. The second frequent flaw was lack of publication bias assessment, in five systematic reviews [15–19]. Two SRS [19, 22] did not present the list of excluded studies and the reason for exclusion and two [15, 25] did not use appropriate methods for the statistical combination when performing a meta-analysis.

**Table 2:**
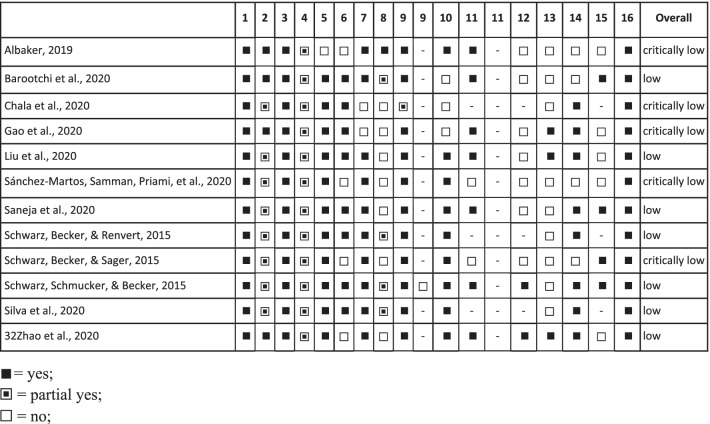
Assessment of methodological quality of the included systematic reviews using AMSTAR2

### Description of primary studies included in SRs

**Fig. 2:**
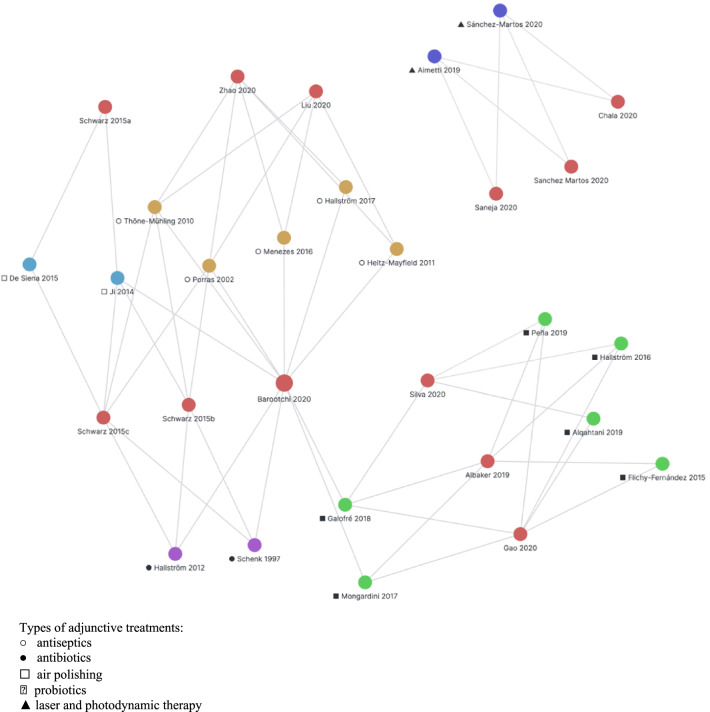
Network of included systematic reviews and primary studies. The systematic reviews and the primary studies were represented by nodes. Each systematic review was linked to the primary studies that were part of it. Types of adjunctive treatments: open circle antiseptics, filled circle antibiotics, open square air–polishing, filled square probiotics, filled triangle laser and photodynamic therapy

The included SRs assessed clinical studies published between 2014 and 2020, including 17 clinical trials (CTs):16 randomized controlled clinical trials (RCTs) and one controlled clinical trial (CCT) [27]. Twelve SRs included two to ten relevant CTs. Some of these 17 individual studies were included in more than one systematic review. The overlaps in the CTs among the SRs are presented as a citation network in Fig. 2. The summary of the outcome, meta-analysis and vote-counting is presented in Table 3, grouped by type of the adjunctive treatment. The characteristics of the primary studies and overlapping among the systematic reviews are presented grouped by type of adjunctive treatment in Additional file 1: Table S3–S7.

**Table 3:** Result summary of systematic reviews of adjunctive antiseptic treatments

Author, year Title	Review characteristics^a^	Review finding^b^						Conclusion
*Antiseptic treatments*								
Barootchi et al., 2020 Nonsurgical treatment for peri-implant mucositis: A systematic review and meta-analysis	5 RCTs (250) AMSTAR2: low	*Meta-analysis*						*Clinical parameter (unspecified)* No additional benefit from the adjunctive treatments (100%) PD MA shows no significant differences between the test and control groups in PD reduction CAL MA shows no significant differences between the test and control groups in CAL improvement
		Outcomes	Studies	Certainty (GRADE)	Effect size			
					Type	Value (CI)	p-value§	
		PD	3 RCTs	(NR)	WMD	− 0.07 [− 0.33, 0.20]	P = 0.62	
		ATL	2 RCTs	(NR)		− 0.13 [− 0.60, 0.35]	P = 0.6	
		Clinical parameter (unspecified) - 5/5 studies reported no significant difference between tests and controls						
Liu et al., 2020 Does chlorhexidine improve outcomes in non-surgical management of peri-implant mucositis or peri-implantitis?: a systematic review and meta-analysis	4 RCTs (212) AMSTAR2: low	Meta-analysis						PD No additional benefit from the adjunctive treatments (100%) MA shows no significant differences between the test and control groups in PD reduction BOP No additional benefit from the adjunctive treatments (100%) CAL Inconclusive (available studies less than three)
		Outcomes	Studies	Certainty (GRADE)	Effect size			
					Type	Value (CI)	p-value§	
		PD	4 RCTs	(NR)	SMD	0.11 [− 0.16, 0.38]	P = 0.42	
		PD - 4/4 studies† reported no significant difference between tests and controls BOP - 4/4 studies reported no significant difference between tests and controls CAL - 1/1 study reported a significant difference favoured test						
(linked systematic reviews) Schwarz, Becker, & Sager, 2015 Efficacy of professionally administered plaque removal with or without adjunctive measures for the treatment of peri‐implant mucositis. A systematic review and meta‐analysis Schwarz, Schmucker, & Becker, 2015 Efficacy of alternative or adjunctive measures to conventional treatment of peri-implant mucositis and peri-implantitis: A systematic review and meta-analysis	2 RCTs (64) AMSTAR2: critically low, low	PD - 1/2 study reported significant differences favoured test - 1/2 studies reported no significant difference between tests and controls BOP - 2/2 studies reported no significant difference between tests and controls GI - 1/1 study reported no significant difference between tests and controls mBI - 1/1 study† reported no significant difference between tests and controls Microbiological - 1/1 study reported no significant difference between tests and controls						Inconclusive (available studies less than three)
Zhao et al., 2020 Clinical efficacy of chlorhexidine as an adjunct to mechanical therapy of peri-implant disease: A systematic review and meta-analysis	5 RCTs (250) AMSTAR2: low	Meta-analysis						BOP No additional benefit from the adjunctive treatments (80%) MA shows no significant differences between the test and control groups in BOP reduction PD MA shows no significant differences between the test and control groups in PD reduction CAL MA shows no significant differences between the test and control groups in CAL improvement
		Outcomes	Studies	Certainty (GRADE)	Effect size			
					Type	Value (CI)	p-value§	
		BOP (reduction in 1 month)	2 RCTs	low	MD	0.10 [− 0.6, 0.25]	P = 0.21	
		BOP (reduction in 3–4 months)	3 RCTs	moderate	MD	0.06 [− 0.03, 0.15]	P = 0.19	
		BOP (reduction in 6–8 months)	2 RCTs	moderate	MD	0.05 [− 0.04, 0.13]	P = 0.30	
		PD (reduction in 1 month)	3 RCTs	moderate	MD	− 0.16 [− 0.38, 0.06]	P = 0.16	
		PD (reduction in 3–4 months)	4 RCTs	moderate	MD	0.02 [− 0.17, 0.20]	P = 0.86	
		PD (reduction in 6–8 months)	2 RCTs	moderate	MD	0.10 [− 0.07, 0.27]	P = 0.24	
		CAL (reduction in 1 month)	2 RCTs	moderate	MD	− 0.24 [− 0.69, 0.20]	P = 0.29	
		CAL (reduction in 3–4 months)	2 RCTs	moderate	MD	− 0.20 [− 0.77, 0.38]	P = 0.50	
		BOP - 4/5 studies reported no significant difference between tests and controls - 1/5 study reported significant improvement in the test groups						
*Probiotic treatment*								
Albaker, 2019 The Effect of Probiotic Administration in the Treatment of Peri-implant Diseases: A Systematic Review and Meta-analysis	5 RCTs (195) AMSTAR2: critically low	Meta-analysis						PD, BOP, PI No additional benefit from the adjunctive treatments (100%) MA shows no significant differences between the test and control groups in PD, BOP and PI reduction
		Outcomes	Studies	Certainty (GRADE)	Effect size			
					Type	Value (CI)	p-value§	
		PPD	4 RCTs	(NR)	WMD	− 0.11 [− 0.43, 0.21]	0.50	
		BOP	2 RCTs	(NR)	OR	1.03 [0.40, 2.62]	0.94	
		PI	2 RCTs	(NR)	OR	0.8 [0.29, 2.18]	0.66	
		PD, - 3/3 studies‡ reported no significant difference between tests and controls BOP - 4/4 studies‡ reported no significant difference between tests and controls PI - 5/5 studies‡ reported no significant difference between tests and controls						
Barootchi et al., 2020 Nonsurgical treatment for peri-implant mucositis: A systematic review and meta-analysis	2 RCTs (62) AMSTAR2: low	PD - 1/1 study† reported no significant difference between tests and controls BOP - 2/2 studies† reported no significant difference between tests and controls PI - 2/2 studies​​† reported no significant difference between tests and controls Total bacteria load - 1/1 study ​† reported no significant difference between tests and controls						Inconclusive (available studies less than three)
Gao et al., 2020 Does Probiotic Lactobacillus Have an Adjunctive Effect in the Nonsurgical Treatment of Peri-Implant Diseases? A Systematic Review and Meta-analysis	6 RCTs (221) AMSTAR2: critically low	Meta-analysis						PD, BOP, PI MA shows no significant differences between the test and control groups in PD, BOP and PI reduction
		Outcomes	Studies	Certainty (GRADE)	Effect size			
					Type	Value (CI)	p-value§	
		PPD (immediately after treatment)	4 RCTs	moderate	MD	− 0.05 [− 0.28, 0.18]	0.67	
		PPD (≥ 2 months after treatment)	5 RCTs	low	MD	− 0.17 [− 1.01, 0.67]	0.69	
		BOP (immediately after treatment)	4 RCTs	moderate	OR	0.75 [0.36, 1.56]	0.44	
		BOP (≥ 1 months after treatment)	4 RCTs	moderate	SMD	0.77 [0.38, 1.56]	0.47	
		PI (immediately after treatment)	5 RCTs	moderate	SMD	− 0.03 [− 0.38, 0.31]	0.85	
		PI (≥ 1 months after treatment)	4 RCTs	moderate	SMD	− 0.37 [− 0.76, 0.02]	0.06	
Silva et al., 2020 Effect of Adjunctive Probiotic Therapy on the Treatment of Peri-implant Diseases–A Systematic Review	4 RCTs (201) AMSTAR2: low	PD - 4/4 studies† reported no significant difference between tests and controls BOP - 2/2 studies†‡ reported no significant difference between tests and controls PI - 4/4 studies‡ reported no significant difference between tests and controls						PD, BOP No additional benefit from the adjunctive treatments (100%) PI No additional benefit from the adjunctive treatments (100%)
*Air-polishing treatment*								
Barootchi et al., 2020 Nonsurgical treatment for peri-implant mucositis: A systematic review and meta-analysis	1 RCT (33) AMSTAR2: low	Clinical parameter (unspecified) - 1/1 study reported no additional clinical benefits of adjunctive glycine powder air polishing PD, BI, PI - 1/1 study reported no significant difference between tests and controls						Inconclusive (available studies less than three)
(linked systemaic reviews) Schwarz, Becker, & Renvert, 2015 Efficacy of air polishing for the non‐surgical treatment of peri‐implant diseases: A systematic review Schwarz, Schmucker, & Becker, 2015 Efficacy of alternative or adjunctive measures to conventional treatment of peri-implant mucositis and peri-implantitis: A systematic review and meta-analysis	1 RCT (33) 21 CCT (30 patients) AMSTAR2: low	PD - 1/2 study reported no significant difference between tests and controls - 1/2 study reported significant differences favoured test BI - 1/2 study reported no significant difference between tests and controls - 1/2 study reported significant differences favoured test						Inconclusive (available studies less than three)
Schwarz, Becker, & Sager, 2015 Efficacy of professionally administered plaque removal with or without adjunctive measures for the treatment of peri‐implant mucositis. A systematic review and meta‐analysis	1 RCT (33) AMSTAR2: critically low	Clinical parameter (unspecified) - 1/1 study reported limited efficacy of the adjunctive glycine powder air polishing PD, BI - 1/1 study reported no significant difference between tests and controls						Inconclusive (available studies less than three)
*Laser and photodynamic treatment*								
Chala et al., 2020 Adjunctive Use of Lasers in Peri-Implant Mucositis and Peri-Implantitis Treatment: A Systematic Review	2 RCTs (288) AMSTAR2: critically low	Clinical parameter (unspecified) - 1/2 study reported no significant clinical benefits of adjunctive use of lasers compared to conventional treatments - 1/2 study reported the benefit of adjunctive use of lasers in reducing bleeding on probing PD - 1/1 study† reported no significant difference between tests and controls BOP - 1/2 study† reported no significant difference between tests and controls - 1/2 study reported a significant difference favoured test PI - 1/1 study† reported no significant difference between tests and controls						Inconclusive (available studies less than three)
Sánchez-Martos, Samman, Priami, et al., 2020 The diode laser as coadjuvant therapy in the non-surgical conventional treatment of peri-implant mucositis: A systematic review and meta-analysis	2 RCTs (288) AMSTAR2: critically low	PD - 2/2 studies reported no significant difference between tests and controls BOP - 1/2 study no significant difference between tests and controls after 3 months - 1/2 study reported significant differences favoured test after 3 months PI - 2/2 studies reported no significant difference between tests and controls Meta-analysis (There was an insufficient presentation of information about the meta-analysis.)						Inconclusive (available studies less than three)
Saneja et al., 2020 Efficacy of different lasers of various wavelengths in treatment of peri-implantitis and peri-implant mucositis: A systematic review and meta-analysis	2 RCTs (288) AMSTAR2: low	Outcomes	Studies	Certainty (GRADE)	Effect size			PD MA shows no significant differences between the test and control groups in PD reduction
					Type	Value (CI)	p-value§	
		PD	2 RCTs	(NR)	MD	− 0.10 [− 0.18, − 0.02]	0.02	
		PD - 2/2 studies reported no significant difference between tests and controls						
*Antibiotic treatment*								
Barootchi et al., 2020 Nonsurgical treatment for peri-implant mucositis: A systematic review and meta-analysis	2 RCTs (69) AMSTAR2: low	Clinical parameter (unspecified) - 1/2 study reported slightly better results in the local antibiotic group with no statistical significance - 1/2 studies reported no additional clinical and microbiological benefits of adjunctive systemic antibiotics PD - 1/1 study reported a greater reduction of PD in the antibiotic group.* BOP - 2/2 study reported greater BOP reduction in the test group.* BI - 1/1 study reported a greater reduction of BI in the antibiotic group.* PI - 1/1 study reported a greater reduction of PI in the antibiotic group.* Microbiological - 1/1 study reported no significant difference in the bacterial counts for all bacterial species between tests and controls						Inconclusive (available studies less than three)
**(**linked systematic reviews) Schwarz, Becker, & Sager, 2015 Efficacy of professionally administered plaque removal with or without adjunctive measures for the treatment of peri‐implant mucositis. A systematic review and meta‐analysis Schwarz, Schmucker, & Becker, 2015 Efficacy of alternative or adjunctive measures to conventional treatment of peri-implant mucositis and peri-implantitis: A systematic review and meta-analysis	2 RCTs (69) AMSTAR2: critically low, low	Clinical parameter (unspecified) - 1/1 study reported no significant differences between antibiotic and control groups for all clinical and microbiological parameters BOP - 1/1 study reported BOP reduction in the antibiotic group, while BOP increased in the control group.*						Inconclusive (available studies less than three)

RCT—randomized controlled clinical trial; PD—probing depth, BOP—bleeding on probing, BI—bleeding index, mBI—modified bleeding index, PI—plaque index, CAL—clinical attachment level; MD—mean difference; NR—not reported

^a^These tables only report parts of the characteristic of included primary studies and quality assessment of the systematic reviews (AMSTAR2). The characteristic of the population, intervention/comparisons, outcomes and quality assessment of the included primary studies are summarized in Tables S3–S7

^b^Review finding is a summary of the information reported in the systematic reviews. The general effectiveness (by studies) is reported on the top, followed by the effectiveness of each parameter. The comparison between test and control groups are in bold. An asterisk indicates the result with the test of statistical significance at the end

^c^Conclusion is provided when there are results from at least three studies available. The first conclusion is about the effectiveness of the treatment with adjunctive when comparing between baseline and last follow-up. The second conclusion is about the effect of adjunctive treatments when comparing between test and control groups. The third conclusion is the report of meta-analysis between test and control groups

^*^The result without the test of statistical significance

^†^The additional information was from the original sources (the primary studies) since the systematic reviews’ information was unspecific

^‡^The data provided in the systematic reviews were corrected by consulting with the original sources (the primary studies)

^§^p-value of the test of the overall effect

The risk of bias of the clinical trials varied from low to high. The SRs used different tools to assess included CTs. Most used the first version of the Cochrane risk-of-bias tool [28, 29]. One [19] used the updated version 30 and one used its original criteria. Notably, the risk of bias assigned to these individual primary studies differed between systematic reviews even if the grading system was the same.

### Interventions and comparators in included primary studies

The included SRs reviewed five RCTs [31–35] regarding adjunctive antiseptic treatment. Chlorhexidine gluconate was used in all trials in the form of gel, solution or spray with different concentrations: 0.12%, 0.2%, 0.5%. The application period differed from 10 days to 12 weeks among those studies.

Six RCTs [36–41] of adjunctive probiotic treatment were identified. Most studies use probiotics containing *Lactobacillus reuteri* in lozenges, which were dissolved in the mouth. Only one study40 used probiotics containing *Lactobacillus brevis* and *Lactobacillus plantarum*. This study also provided a probiotic mixture applied in the peri-implant sulcus in the clinic before letting the patients continue with the lozenges. The administration time for the probiotic lozenges varied from 3 weeks to 3 months.

The studies of air-polishing included one randomized controlled clinical trial [42] and one controlled clinical trial [43]. Both trials experimented with a glycine powder air-polishing device by applying at a submucosal level for five seconds on each affected implant site. Two RCTs [44, 45] assessed adjunctive laser and photodynamic treatment. They both used a diode laser in pulse mode by applying for 30 s per surface; however, the wavelength and power settings differed between the two studies. Two adjunctive antibiotic treatment RCTs [46, 47] were reviewed. One study assessed the systemic antibiotic Azithromycin, prescribed for 5 days [46]. Another study evaluated local antibiotic therapy, applying tetracycline HCl fibres in the peri-implant sulcus for ten days [47].

All the controlled trials compared the adjunctive treatment with NSMD (using either hand or ultrasonic instrument), polishing (using polishing paste and rubber cup), or both. However, some studies include adjunctive treatments in their conventional treatment protocol. One of the probiotic treatment studies [40] had photodynamic therapy as part of the control treatment. Some studies included peri-implant sulcus irrigation using 3% hydrogen peroxide [44], 0.12% CHX + 0.05% CPC45, or 0.12% CHX mouth rinsing [47] in their control treatment.

### Data analysis in included SRs

All included SRs summarized the data of the individual studies and provided a narrative conclusion regarding the effectiveness of the adjunctive treatments. However, six SRs [16–19, 23, 24] performed MA of effects for additional benefits of the adjunctive treatment for peri-implant mucositis. These MAs showed no significant difference in probing depth, bleeding on probing, clinical attachment level, or plaque index outcomes between control (conventional treatment or NSMD) and test (conventional treatment with adjunctive therapy) groups. Four SRs [20–22, 45] did not conduct MA owing to heterogeneity present in the clinical trials concerning population (i.e., dental implant and restoring unit, peri-implant case definition), adjunctive treatment protocol, conventional treatment protocol, and outcome measurement (i.e., clinical parameters and follow-up period for evaluation). Two SRs [25, 26] conducted the MA of similar clinical outcomes parameters (bleeding on probing, gingival index and probing depth) of different types of adjunctive treatment (antibiotic, antiseptic, air-polishing and probiotic treatment); therefore, the present umbrella review analyzed the MA outcomes of these two SRs [25, 26].

## Discussion

This umbrella review included 12 systematic reviews to examine the effect of adjunctive measures on PIM treatment. Considering PICOS framework, not all of the included reviews established definite and narrow PICOS frameworks. There was also variability in PICOS elements (i.e., population, intervention and comparators, outcomes, and study types) among the included systematic reviews.

The population and intervention elements were not well specified in most of the included systematic reviews. The focused populations had both PIM and peri-implantitis and intervention included all types of adjunctive treatments reported in literature. For instance, two systematic reviews [24, 25] included studies of peri-implant mucositis exclusively; however, they did not specify the types of the studied intervention.

Considering case definition of PIM, most systematic reviews did not specify the diagnostic criteria. The inclusion of the studies of peri-implant mucositis was based on the diagnosis assigned in the respective publications. Only one systematic review [45] referred to the 2017 World Workshop classification [48]. Most SRS used more than one parameter to assess the treatment outcome. The most studied outcome was probing depth, followed by bleeding on probing, plaque index and clinical attachment level.

There were differences between the control treatments among the individual studies. While most studies had NSMD as conventional treatment, some also added antiseptic treatment [36, 41, 44, 45, 47] or photodynamic treatment [40] in their control treatments. In addition, there were discrepancies in NSMD protocol, as included studies used curettes or ultrasonic devices, rubber cups and polishing paste, or both.

Some SRs also specified a minimum follow-up time of 1 month [18, 20] or 3 months [15, 22]. The follow-up period of the included primary studies ranged from 1 to 8 months. Most of the studies presented no significant difference between the test and control groups throughout the period of their follow-up. Only two primary studies regarding adjunctive antiseptic treatments [31, 34] showed significant differences that favored the test groups in the short term of the first 3 months; however, the studies did not continue the follow-up to see whether the effect would persist in a longer follow-up period.

Three SRs [24–26] (SRs) reviewed different adjunctive treatments and concluded that there was no additional benefit in the adjunctive treatment of PIM when compared to NSMD. Five SRs which reviewed antiseptics [16, 17] air-polishing [21], probiotics [20], and laser and photodynamic treatment [22] also concluded that the adjunctive treatment was not superior to conventional treatment. Four SRs regarding probiotics [18, 19] and laser and photodynamic therapy [15, 23] suggested that the benefit of adjunctive treatment was inconclusive and called for further clinical trials.

Three SRs regarding antiseptic treatment that performed MA indicated no significant difference in probing depth [16, 17, 24] bleeding on probing [17], and clinical attachment level [17, 24] between groups of conventional treatment and adjunctive treatment. MA for effects of adjunctive probiotic treatment was carried out in two SRs [18, 19].and noted no significant difference between conventional and adjunctive treatment groups in probing depth, bleeding on probing, and plaque index outcomes. Only one SR [23] of adjunctive laser and photodynamic treatment conducted MA. Adjunctive laser therapy did not significantly differ in probing depth from conventional treatment.

The effectiveness of the adjunctive treatments presented in the SRs was further determined by vote-counting based on a statistically significant difference in comparison of clinical parameters. Adjunctive antiseptic treatment shows no additional benefit in improving probing depth 16 and bleeding on probing [16, 17]. There was also no additional benefit of adjunctive probiotic treatment in improving probing depth, bleeding on probing, and plaque index [18, 20]. The effectiveness of the other adjunctive treatments could not be synthesized by vote-counting as the SRs included less than three primary studies.

The included SRs' overall confidence (AMSTAR 2) ranged from low to critically low. Overall, the summarized evidence indicated that adjunctive treatments did not significantly improve the clinical outcome parameters compared to NSMD.

Despite a rigorous methodology, this umbrella review has limitations. Firstly, the included systematic reviews and clinical trials were not of high quality and were few in number, including 12 SRs and 17 primary studies. About two-thirds of the primary studies presented with a high risk of bias. The confidence of the SRs was also low to critically low, according to the AMSTAR 2 assessment. Furthermore, the included SRs analyzed overlapping primary studies, which could account for their consistent findings. Finally, the present umbrella review opted for a non-statistical approach in data synthesis by implementing the vote-counting method to identify adjunctive treatment effectiveness for each clinical parameter. However, this approach has limitations [30] as vote-counting does not consider the effect size and the precision of the statistical estimate of the primary studies. Systematic reviews with a narrow scope were lacking and this umbrella review also demonstrated a lack of randomized controlled clinical trials. Sufficient RCTs of good quality need to be available to enable systematic reviews with a clear and narrow scope.

While the conclusion of this umbrella review does not support the general use of adjunctive treatment in managing PIM, patient subsets that may receive benefit from these therapies remain an open question. Clinical trials in patients with a history of periodontitis, diabetes or smoking with increased risk for peri-implant diseases are warranted. In addition, the adjunctive treatment for implants with local risk indicators may be considered [49]. PIM around the deep mucosal tunnel implant presents delayed disease resolution after non-surgical debridement [50]. Implant design with an over-contour prosthetic profile also could pose risks for peri-implant health [51, 52]. The role of adjunctive treatments in such situations needs further investigation. Furthermore, there are several reported adjuncts to NSMD for peri-implant disease including ozone therapy [53, 54], desiccant application [55], electrolytic cleaning procedures [56] and herbal medications [57] for which no evidence was synthesized in the systematic reviews of peri-implant mucositis included in the present study. Therefore, conclusions regarding the efficacy of these measures cannot be drawn from the present study. More clinical trials and subsequent SRs are warranted in order to clarify the effectiveness of emerging therapies.

## Conclusion

A small number of primary studies and SRs address outcomes of adjunctive treatment for peri-implant mucositis and the quality of available SRs is generally low. Most of the primary studies have a high risk of bias, with discrepancy in the outcome measurements and follow-up times reported. Within these limitations, the present umbrella review failed to show significant benefit from adjunctive treatments to improve the outcome of NSMD in PIM and no specific adjunctive therapies have emerged as clearly superior to NSMD, so far.

## Supplementary Information

**Additional file 1: Table S1.** Eligibility criteria form. **Table S2.** Excluded reviews at the assessment of full-text and the main reasons for exclusion. **Table S3.** Characteristic of primary studies and overlap amongst systematic reviews of adjunctive antiseptic treatment. **Table S4.** Characteristic of primary studies and overlap amongst systematic reviews of adjunctive probiotic treatment. **Table S5.** Characteristic of primary studies and overlap amongst systematic reviews of adjunctive air-polishing treatment. **Table S6.** Characteristic of primary studies and overlap amongst systematic reviews of adjunctive laser and photodynamic treatment. **Table S7.** Characteristic of primary studies and overlap amongst systematic reviews of adjunctive local and systemic antibiotic treatment. **Additional document 1.** Data items of the systematic reviews and primary studies**.**

## Data Availability

Data will be shared upon reasonable request.
