# Supplementary material for: The effectiveness of adjunctive measures in managing peri-implant mucositis: an umbrella review
Source: Int J Implant Dent. 2022 Jun 8;8:26. doi: 10.1186/s40729-022-00426-2 (PMC9177933; doi:10.1186/s40729-022-00426-2)
Supplement: Supplementary file 1 — Additional file 1: Table S1. Eligibility criteria form. Table S2. Excluded reviews at the assessment of full-text and the main reasons for exclusion. Table S3. Characteristic of primary studies and overlap amongst systematic reviews of adjunctive antiseptic treatment. Table S4. Characteristic of primary studies and overlap amongst systematic reviews of adjunctive probiotic treatment. Table S5. Characteristic of primary studies and overlap amongst systematic reviews of adjunctive air-polishing treatment. Table S6. Characteristic of primary studies and overlap amongst systematic reviews of adjunctive laser and photodynamic treatment. Table S7. Characteristic of primary studies and overlap amongst systematic reviews of adjunctive local and systemic antibiotic treatment. Additional document 1. Data items of the systematic reviews and primary studies. [file 40729_2022_426_MOESM1_ESM.docx]

# Additional tables

## Table S1: Eligibility criteria form

| Citation (Author year): | | | |
| --- | --- | --- | --- |
| **1. Study Design: a systematic review with or without meta-analysis** | | | |
| Did the review implement a systematic search for primary studies? | Yes | No | Unclear |
| **2. Population: adult patients with the diagnosis of  peri-implant mucositis** | | | |
| 2.1 Did the review include the primary studies of adult patients with the diagnosis of peri-implant mucositis? | Yes | No | Unclear |
| 2.2 Does the data of the population of interest can be extracted from the review that does not include the patients with peri-implant mucositis exclusively? | Yes | No | Unclear |
| **3. Intervention: non-surgical professional debridement procedures with adjunctive interventions** | | | |
| 3.1 Did the review include the primary studies of which the interventions are considered of non-surgical professional debridement procedures with adjunctive interventions?  ◻ laser therapy,  ◻ photodynamic therapy,  ◻ air polishing,  ◻ local antiseptic application,  ◻ local or systemic antibiotic application | Yes | No | Unclear |
| 3.2 Does the data of the adjunctive interventions of interest can be extracted from the review that does not include one type of adjunctive treatment exclusively? | Yes | No | Unclear |
| **4. Comparison: non-surgical professional debridement procedures alone** | | | |
| Did the review include the primary studies of which the interventions are considered of non-surgical professional debridement procedures alone? | Yes | No | Unclear |
| **5. Outcomes: clinical, microbiological and immunological parameters** | | | |
| Did the review report quantitative improvements on at least one outcome, i.e. clinical (probing and visual assessment), microbiological and immunological parameters? | Yes | No | Unclear |
| **6. Language: English** | | | |
| Was the review published the full text in English? | Yes | No | Unclear |
| **Final decision: eligible for inclusion** |  | | |
| Should this review be eligible for inclusion?  Yes, if all answers above are yes.  No, if there is at least one “No” answer.  Unclear, if there is at least one “Unclear” answer. | Yes | No | Unclear |

## Table S2: Excluded reviews at the assessment of full-text and the main reasons for exclusion

| **Author, year of publication** | **The main reason for exclusion** |
| --- | --- |
| Albaker, ArRejaie, Alrabiah, & Abduljabbar (2018) | All five included primary studies did not fulfil the eligibility criteria when assessing against PICO elements of the systematic review and this umbrella review. |
| Arbildo-Vega (2021) | The full article could not be obtained after requesting the journal and contacting the corresponding author. |
| Ata-Ali et al. (2015) | The review didn’t have a well-constructed PICO framework; hence, the study selection was carried out in an unsystematic fashion. |
| Javadi (2017) | The review is not a systematic review. It was identified as a review with a systematic search. |
| Khouly et al. (2020) | This review studied growth factors for peri-implant mucositis treatment. The adjunctive treatments for peri-implant mucositis considered by the team of this umbrella review do not include the use of growth factors. |
| Lin et al. (2018) | All three included primary studies did not fulfil the eligibility criteria when assessing PICO elements against this umbrella review. The author also concluded that the systematic review was not able to warrant any answer about non-surgical treatment for peri-implant mucositis. |
| Shahmohammadi (2021) | The review did not present and summarise the data comparing effectiveness among treatments of peri-implant mucositis. The meta-analysis did not perform for peri-implant mucositis exclusively. |
| Suárez-López Del Amo et al. (2016) | The PICOS framework of the review does not agree with that of the present umbrella review. No results about peri-implant mucositis could be extracted from this review. |
| Vohra et al. (2014) | The review didn’t have a well-constructed PICO framework; hence, the study selection was carried out in an unsystematic fashion. |
| Zeza & Pilloni (2012) | The PICOS framework of the review does not agree with that of the present umbrella review. |

## Table S3: Characteristic of primary studies and overlap amongst systematic reviews of adjunctive antiseptic treatment

| **Systematic reviews** | **Primary studies** | | | | | **Meta-analysis** | **Quality of evidence** |
| --- | --- | --- | --- | --- | --- | --- | --- |
|  | Hallström et al., 2017 | Heitz-Mayfield et al., 2011 | Menezes et al., 2016 | Porras et al., 2002 | Thöne-Mühling et al., 2010 |  |  |
| Barootchi et al., 2020 | ◇ | ◇ | ◆ | ◆ | ◆ | Yes | low |
| Liu et al., 2020 |  | ◆ | ◆ | ◆ | ◆ | Yes | low |
| Schwarz, Becker, & Sager, 2015 |  |  |  | ◇ | ◇ |  | critically low |
| Schwarz, Schmucker, & Becker, 2015 |  |  |  | ◇ | ◇ |  | low |
| Zhao et al., 2020 | ◇ | ◆ | ◇ | ◇ | ◆ | Yes | low |
| **Risk of Bias**  (number of  systematic reviews) | Unclear (1)  High (1) | Unclear (2)  High (1) | Unclear (2)  High (1) | Unclear (1)  High (4) | Unclear (1)  High (4) |  |  |
| Characteristic of the primary studies | | | | | | |  |
| Study type  (number of implants) | RCT  (38 implants) | RCT (29 implants) | RCT  (119 implants) | RCT  (28 implants) | RCT  (36 implants) |  |  |
| Peri-implant mucositis case definition | **Probe**: bleeding, pus  **PD**: ≥ 4 mm  **RBL**: ≤ 2mm | **Probe**: bleeding  **PD**: -  **RBL**: 0 | **Probe**: bleeding  **PD**: ≤ 5 mm  **RBL**: ≤ 2 threads | **Probe**: bleeding  **PD**: ≤ 5 mm  **RBL**: “incipient lesion” | **Probe**: bleeding  **PD**: -  **RBL**: 0 |  |  |
| Systemic disorders | excluded poorly control diabetes | (not specified in text) | (not specified in text) | excluded | excluded |  |  |
| Smokers | included | included | excluded | excluded | included |  |  |
| Treated periodontitis | (not specified in text) | included | (not specified in text) | excluded | included |  |  |
| Adjunctive treatment  (test group) | 0.2% CHX gel  brushing once daily  for 12 wk | 0.5% CHX gel  brushing twice daily  for 4 wk | 0.12% CHX solution, tongue brushing, rinsing, subgingival irrigation, once after debridement  0.12% CHX solution  rinsing twice daily  for 2 wk | 0.12% CHX solution, subgingival irrigation, once after debridement  0.12% CHX solution  rinsing twice daily  for 10 days | 0.2% CHX solution  rinsing once daily  for 2 wk  0.2% CHX solution  spray tonsils once daily for 2 wk |  |  |
| Common treatment  (control and test group) | mechanical debridement (titanium curettes)  +  polishing  (rubber cup and polishing paste) | mechanical debridement (titanium-coated/ carbon fibre curettes)  +  polishing  (rubber cup and polishing paste) | mechanical debridement  (plastic curettes) | mechanical debridement  (plastic curettes)  +  polishing  (rubber cup and polishing paste) | mechanical debridement  (plastic curettes and polyetheretherketone-coated ultrasonics  instruments) |  |  |
| Follow-up period | 3 months | 3 months | 6 months | 3 months | 8 months |  |  |

◇ = the primary study was included in the systematic review

◆  = the primary study was included in the systematic review and meta-analysis

RCT = randomised controlled clinical trials; PD = probing depth; RBL = radiographic bone loss

## Table S4: Characteristic of primary studies and overlap amongst systematic reviews of adjunctive probiotic treatment

| **Systematic reviews** | **Primary studies** | | | | | | **Meta- analysis** | **Quality of evidence** |
| --- | --- | --- | --- | --- | --- | --- | --- | --- |
|  | Alqahtani et al., 2019 | Flichy-Fernández et al., 2015 | Galofré et al., 2018 | Hallström et al., 2016 | Mongardini et al., 2017 | Peña et al., 2019 |  |  |
| Albaker, 2019 |  | ◆ | ◆ | ◆ | ◇ | ◆ | Yes | Critically low |
| Barootchi et al., 2020 |  |  | ◇ |  | ◇ |  |  | low |
| Gao et al., 2020 | ◆ | ◆ | ◆ | ◆ | ◇ | ◆ | Yes | Critically low |
| Silva et al., 2020 | ◇ |  | ◇ | ◇ |  | ◇ |  | low |
| **Risk of Bias**  (number of  systematic reviews) | Low (2) | Low (1) High (1) | Low (4) | Low (2)  High (1) | Low (3) | Low (2)  High (1) |  |  |
| Characteristic of the primary studies | | | | | | | | |
| Study type  (number of implants) | RCT  (80 implants) | RCT  (45 implants) | RCT  (22 implants) | RCT  (49 implants) | RCT  (20 implants) | RCT  (50 implants) |  |  |
| Peri-implant mucositis case definition | **Probe**: bleeding  **PD**: ≥ 3 mm  **RBL**: ≤ 2mm | **Probe**: bleeding  **PD**: -  **RBL**: 0 | **Probe**: bleeding, pus  **PD**: ≥ 4 mm  **RBL**: 0 | **Probe**: bleeding, pus  **PD**: ≥ 4 mm  **RBL**: > 2mm | **Probe**: -  **PD**: ≤ 4mm  **RBL**: < 2mm | **Probe**: bleeding  **PD**: -  **RBL**: 0 |  |  |
| Systemic disorders | excluded | excluded | excluded | excluded poorly controlled diabetes | excluded | excluded |  |  |
| Smokers | included | excluded | excluded | included | included | included |  |  |
| Treated periodontitis | exclude | (not specified in text) | included | (not specified in text) | included | included |  |  |
| Adjunctive treatment  (test group) | **Strain**: L.reuteri  **Form**: lozenge  one lozenge  twice a day  for 3 weeks | **Strain**: L.reuteri  **Form**: lozenge  one lozenge a day  for 4 weeks | **Strain**: L.reuteri  **Form**: lozenge  one lozenge dissolved in mouth once a day  for4 weeks | **Strain**: L.reuteri  **Form**: lozenge  one lozenge dissolved in mouth twice a day  for 3 months | **Strain**:  L. brevis,                L. plantarum  **Form**: lozenge  probiotic mixture applied into sulcus in the clinic  +  one lozenge a day  for 4 weeks | **Strain**: L.reuteri  **Form**: lozenge  one lozenge a day  for 4 weeks |  |  |
| Common treatment  (control and test group) | mechanical debridement  (ultrasonic instruments)  +  mouth rinsing  (0.12% CHX)  twice daily  for 2 weeks | polishing  (rubber cup and polishing paste) | mechanical debridement  (carbon-tipped ultrasonics, titanium curettes) | polishing  (rubber cup and polishing paste) | polishing  (brush, rubber cup and polishing paste)  +  photodynamic therapy | mechanical debridement (titanium-tipped ultrasonics)  +  mouth rinsing  (0.12% CHX)  twice daily  for 2 weeks |  |  |
| Follow-up period | 6 months | 1 month | 3 month | 6 months | 6 weeks | 4 months |  |  |

◇ = the primary study was included in the systematic review

◆  = the primary study was included in the systematic review and meta-analysis

RCT = randomised controlled clinical trials; PD = probing depth; RBL = radiographic bone loss

## Table S5: Characteristic of primary studies and overlap amongst systematic reviews of adjunctive air-polishing treatment

| **Systematic reviews** | **Primary studies** | | **Meta-analysis** | **Quality of evidence** |
| --- | --- | --- | --- | --- |
|  | De Siena et al., 2015 | Ji et al., 2014 |  |  |
| Barootchi et al., 2020 |  | ◇ | No | low |
| Schwarz, Becker, & Renvert, 2015 | ◇ | ◇ | No | low |
| Schwarz, Becker, & Sager, 2015 |  | ◇ | No | Critically low |
| Schwarz, Schmucker, & Becker, 2015 | ◇ | ◇ | No | low |
| **Risk of Bias**  (number of systematic reviews) | non-assessed | High (4) |  |  |
| Characteristic of the primary studies | | | |  |
| Study type  (number of implants) | CCT  (30 patients) | RCT  (33 implants) |  |  |
| Peri-implant mucositis case definition | **Probing**: bleeding  **Probing depth**: -  **Radiographic bone loss**: ≤ 3mm | **Probing**: bleeding  **Probing depth**: ≥ 4 mm  **Radiographic bone loss**: 0 mm |  |  |
| Systemic disorders | (not specified in text) | excluded |  |  |
| Smokers | (not specified in text) | (not specified in text) |  |  |
| Treated periodontitis | (not specified in text) | (not specified in text) |  |  |
| Adjunctive treatment  (test group) | glycine powder air-polishing device  submucosal application  for 5 sec on each site | glycine powder air-polishing device was inserted submucosal application  for 5 sec on each site |  |  |
| Common treatment  (control and test group) | mechanical debridement  (Teflon curettes)  +  polishing | mechanical debridement  (carbon-tipped ultrasonic scaler) |  |  |
| Follow-up period | 6 months | 3 months |  |  |

◇ = the primary study was included in the systematic review

RCT = randomised controlled clinical trials; CCT = controlled clinical trials 

## Table S6: Characteristic of primary studies and overlap amongst systematic reviews of adjunctive laser and photodynamic treatment

| **Systematic reviews** | **Primary studies** | | **Meta-analysis** | **Quality of evidence** |
| --- | --- | --- | --- | --- |
|  | Aimetti et al., 2019 | Sánchez-Martos et al., 2020 |  |  |
| Chala et al., 2020 | ◇ | ◇ | No | critically low |
| Sánchez-Martos, Samman, Priami, et al., 2020 | ◇ | ◇ | No | critically low |
| Saneja et al., 2020 | ◆ | ◆ | Yes | low |
| **Risk of Bias**  (number of systematic reviews) | Low (2) | Low (2)  High (2) |  |  |
| Characteristic of the primary studies | | | |  |
| Study type  (number of implants) | RCT  (220) | RCT  (68) |  |  |
| Peri-implant mucositis case definition | **Probing**: bleeding, pus  **Probing depth**: ≥ 4 mm  **Radiographic bone loss**: < 2 mm | **Probing**: bleeding  **Probing depth**: -  **Radiographic bone loss**: 0 |  |  |
| Systemic disorders | excluded | included |  |  |
| Smokers | included | included |  |  |
| Treated periodontitis | included | (not specified in text) |  |  |
| Adjunctive treatment  (test group) | diode laser  wavelength of 980 nm,  power of 2.5  watt  in pulsed mode  30 seconds per surface | diode laser  wavelength of 810 nm,  power of 1 watt  in pulsed mode  30 seconds per surface |  |  |
| Common treatment  (control and test group) | mechanical debridement  (ultrasonic,  titanium-coated or carbon fiber curettes)  +  sulcus irrigation  (3% hydrogen peroxide)   for 10 s | mechanical debridement  (plastic curettes)  +  sulcus irrigation  (0.12% chlorhexidine + 0.05% cetylpyridinium chloride) |  |  |
| Follow-up period | 3 months | 3 months |  |  |

◇ = the primary study was included in the systematic review

◆  = the primary study was included in the systematic review and meta-analysis

RCT = randomised controlled clinical trials

## Table S7: Characteristic of primary studies and overlap amongst systematic reviews of adjunctive local and systemic antibiotic treatment

| **Systematic reviews** | **Primary studies** | | **Meta-analysis** | **Quality of evidence** |
| --- | --- | --- | --- | --- |
|  | Hallström et al., 2012 | Schenk et al., 1997 |  |  |
| Barootchi et al., 2020 | ◇ | ◇ | No | low |
| Schwarz, Becker, & Sager, 2015 | ◇ | ◇ | No | critically low |
| Schwarz, Schmucker, & Becker, 2015 | ◇ | ◇ | No | low |
| **Risk of Bias**  (number of systematic reviews) | Unclear (1) High (2) | High (3) |  |  |
| Characteristic of the primary studies | | | | |
| Study type  (number of implants) | RCT Split-mouth Design (24 implants) | RCT, parallel  (45 implants) |  |  |
| Peri-implant mucositis case definition | **Probing**: bleeding, pus  **Probing depth**: ≥ 4 mm  **Radiographic bone loss**: < 2 mm | **Probing**: bleeding  **Probing depth**: ≥ 4 mm  **Radiographic bone loss**: 0 mm |  |  |
| Systemic disorders | excluded | excluded |  |  |
| Smokers | Non-specified | Non-specified |  |  |
| Treated periodontitis | included | Non-specified |  |  |
| Adjunctive treatment  (test group) | systemic antibiotics  Azithromycin®  500 mg for  day 1  250 mg days 2–4 | local antibiotics  Tetracycline HCl fibres  (a monolithic ethylene-vinyl acetate fibre containing 25% tetracycline hydrochloride)  fibre applied in peri-implant sulcus for 10 days |  |  |
| Common treatment  (control and test group) | mechanical debridement (titanium curettes)  +  polishing  (rubber cup and polishing paste) | mechanical debridement (steel curettes)  +  Polishing (rubber cup)  +  mouth rinsing (0.12% CHX) twice daily for 10 days |  |  |
| Follow-up period | 6 months | 3 months |  |  |

◇ = the primary study was included in the systematic review

RCT = randomised controlled clinical trials

# Additional documents

## Additional document 1: Data items of the systematic reviews and primary studies

### Data items of the systematic reviews

The following data items were extracted to describe the characteristics of the systematic reviews included in this umbrella review.

**Identification**

1. Bibliographic details
   1. The family name of the first author
   2. Year of publication
   3. Publishers (if applicable)
2. Type of source (journal articles, government reports, conference proceedings, thesis)

**Additional details**

1. Peer-review (peer-reviewed, non-peer-reviewed)
2. Country of corresponding authors
3. Funding Source (university, government, industry)

**Methodological and report characteristics**

1. PICOS frameworks
2. PICO questions
3. Search strategy
   1. Number of sources
   2. Last year of the search
4. Population (peri-implant mucositis, both peri-implant mucositis and peri-implantitis)
   1. Case definition of peri-implant mucositis
   2. Restriction of systematic factors (i.e. smoking, radiotherapy, diabetes)
   3. History of periodontitis
5. Interventions (details on interventions)
   1. Mode of treatments (adjunctive treatment, alternative treatment)
   2. Types of adjunctive intervention
6. Comparisons (details on comparisons)
7. Outcome (specific results provided in the reviews)
8. Study design
   1. Types of included primary studies
   2. Number of included primary studies
   3. Types of eligible primary studies
   4. Number of eligible primary studies

**Results and conclusions**

1. The effectiveness of the interested interventions (those with adjunctive intervention)
2. The additional effect of the interested interventions (those with adjunctive intervention)
3. A number of studies reported positive results

### Data items of the primary studies

The following data items were extracted to describe the characteristics of the primary studies of the selected systematic reviews.

**Identification**

1. Bibliographic details
   1. The family name of the first author
   2. Year of publication
   3. Publishers (if applicable)
2. Type of Source (journal articles, government reports, conference proceedings, thesis)

**Additional details**

1. Peer-review (peer-reviewed, non-peer-reviewed)
2. Publication type (subscription-based, open access)
3. Country of corresponding authors
4. Funding source (university, government, industry)

**Methodological and report characteristics**

1. Type of studies (randomised controlled trial, control clinical trials)
2. PICO frameworks
3. PICO questions
4. Population (peri-implant mucositis, both peri-implant mucositis and peri-implantitis)
   1. Case definition of peri-implant mucositis
   2. Restriction of systematic factors (i.e. smoking, radiotherapy, diabetes)
   3. History of periodontitis
5. Interventions (details on interventions)
   1. Mode of treatments (adjunctive treatment, alternative treatment)
   2. Types of adjunctive intervention
6. Comparisons (details on comparisons)
7. Outcome (specific results provided in the reviews)
8. Period of follow-up

**Results and conclusions**

1. Intra-group (test group) comparison (between baseline and the last follow-up)
2. Inter-group comparison (between test and control)
